# A Review of Enteric Methane Emission Measurement Techniques in Ruminants

**DOI:** 10.3390/ani10061004

**Published:** 2020-06-08

**Authors:** Yiguang Zhao, Xuemei Nan, Liang Yang, Shanshan Zheng, Linshu Jiang, Benhai Xiong

**Affiliations:** 1 State Key Laboratory of Animal Nutrition, Institute of Animal Sciences, Chinese Academy of Agricultural Sciences, Beijing 100193, China; zhaoyiguang@caas.cn (Y.Z.); xuemeinan@126.com (X.N.); yangliang@caas.cn (L.Y.); zhengshanshan@caas.cn (S.Z.); 2 Beijing Key Laboratory for Dairy Cow Nutrition, Beijing University of Agriculture, Beijing 102206, China

**Keywords:** greenhouse gas, methane, measurement technique, ruminants

## Abstract

**Simple Summary:**

Enteric methane emissions from ruminants are a major contributor to atmospheric greenhouse gas accumulation in agricultural production systems, which may consequently contribute to climate change. Accurate measurement of methane production in ruminants is vital to develop robust national greenhouse gas emission inventories and to evaluate mitigation strategies of methane emissions. This review summarizes several common methane measurement techniques suitable for ruminant production and discusses the advantages and disadvantages of each method. There is not a perfect technique for all situations. The appropriate technique depends on the objectives and resources available. Sophisticated techniques such as respiration chambers, sulphur hexafluoride tracer and ventilated hood are usually highly accurate but labor and time intensive. Simplified techniques such as GreenFeed, sniffer method, facemask, laser methane detector and portable accumulation chamber may be less accurate but are rapid with a high throughput. In general, an understanding of measurement mechanisms and the advantages and disadvantages with an appreciation of animal behavior and welfare is necessary for all techniques.

**Abstract:**

To identify relationships between animal, dietary and management factors and the resulting methane (CH_4_) emissions, and to identify potential mitigation strategies for CH_4_ production, it is vital to develop reliable and accurate CH_4_ measurement techniques. This review outlines various methods for measuring enteric CH_4_ emissions from ruminants such as respiration chambers (RC), sulphur hexafluoride (SF_6_) tracer, GreenFeed, sniffer method, ventilated hood, facemask, laser CH_4_ detector and portable accumulation chamber. The advantages and disadvantages of these techniques are discussed. In general, RC, SF_6_ and ventilated hood are capable of 24 h continuous measurements for each individual animal, providing accurate reference methods used for research and inventory purposes. However, they require high labor input, animal training and are time consuming. In contrast, short-term measurement techniques (i.e., GreenFeed, sniffer method, facemask, laser CH_4_ detector and portable accumulation chamber) contain additional variations in timing and frequency of measurements obtained relative to the 24 h feeding cycle. However, they are suitable for large-scale measurements under commercial conditions due to their simplicity and high throughput. Successful use of these techniques relies on optimal matching between the objectives of the studies and the mechanism of each method with consideration of animal behavior and welfare. This review can provide useful information in selecting suitable techniques for CH_4_ emission measurement in ruminants.

## 1. Introduction

**Figure 1:**
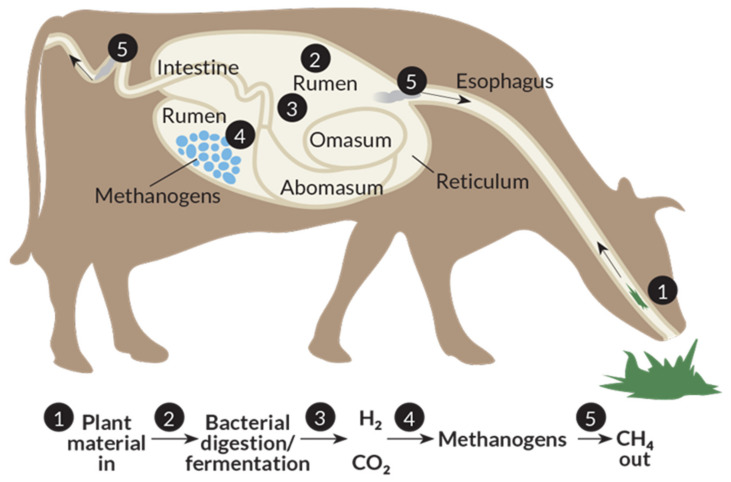
Processes of methane production in ruminants [8].

Greenhouse gas (GHG) emissions contribute to global warming, consequently leading to extreme climate change that affects crop productivity and food security [1,2]. On the other hand, the demand of animal products are expected to increase over time, which requires more livestock to be reared and consequently increases our environmental footprint [3]. The growth in demand has been driven by a number of factors, such as increasing world population, fast urbanization and continuous income growth [4]. Methane (CH_4_) emissions from ruminants, as one of the most important pollutants from agriculture, contribute to the atmospheric GHG accumulation. The atmospheric warming effect of CH_4_ is 28 times as strong as CO_2_ [5]. Approximately 80 million tons of CH_4_ are produced by ruminant livestock production systems each year, representing about 28% of global anthropogenic emission of CH_4_ [6]. CH_4_ is eructated or exhaled mainly through the mouth and nostrils as a byproduct of rumen anerobic fermentation, by which, ruminal microbes digest the feed into absorbable nutrients for the host animal. CH_4_ is predominantly produced by methanogens in the process of anerobic degradation of plant biomass in rumen (Figure 1) [7].

The accurate measurement of CH_4_ emissions from ruminants is crucial to develop a robust inventory, or to develop strategies in mitigating the environmental footprint from animal production systems. CH_4_ production is associated with many factors such as animal growth stage, production efficiency, behavior, feed intake, dietary chemical composition and animal management, etc. [9]. On the other hand, any technique affecting these factors will limit its suitability for CH_4_ measurement. The appropriateness of a technique is highly dependent on the objectives of the measurement and the mechanism of the technique. Nevertheless, an appreciation of animal behavior and welfare is important for all methods. Traditional techniques in quantifying enteric CH_4_ emissions include open-circuit respiration chamber (RC) for individual indoor animals [10,11,12] and sulfur hexafluoride (SF_6_) tracer technique for individual and herd animals, both indoor and outdoor [13,14,15]. Other measurement techniques such as GreenFeed (GF) [16,17], sniffer method [18,19], ventilated hood [20,21], facemask [22,23], laser CH_4_ detector (LMD) [24,25] and portable accumulation chamber (PAC) [26,27] are also reported.

## 2. Measurement Techniques

### 2.1. Respiration Chamber

Respiration chamber is a well-established, well-documented and reliable CH_4_ measurement system, as it is the “gold standard” that accurately measures total CH_4_ production from rumen and hindgut fermentation [28,29,30]. In this technique, an animal is held in a sealed chamber which is large enough to comfortably accommodate them and which is maintained under slightly negative atmospheric pressure. This ensures that any undetected or unavoidable gaseous leaks flow inwards rather than outwards, thereby avoiding any loss of gaseous product [31,32]. Methane emissions are calculated by the measured airflow multiplied by the difference in concentrations between the inlet and outlet air [31,33]. This is facilitated by automated sampling and analysis using an infrared gas analyzer, which repeatedly determines the concentration of CH_4_ in both the inlet and exhaust air. Often, a multi-gas analyzer which integrates the measurements of CH_4_, CO_2_, O_2_ and NH_3_, etc., is used to investigate the GHG emissions and heat production of the animals simultaneously [34,35]. In fact, RC was initially used to quantify gaseous exchange (i.e., O_2_ consumption and CO_2_ and CH_4_ production) to calculate heat production and energy balance in the energy metabolism of animals [36]. Open-circuit RC has replaced the earlier closed-circuit RC previously used by Blaxter and Clapperton [37], providing a relatively simple and accurate way of measuring the heat exchange of animals held within them. Moreover, there are optional sensors for detecting temperature, humidity and pressure installed in some RCs for environmental monitoring [38]. The size of RCs varies according to the species of animals they are intended to be used with. For example, CH_4_ output measurements using RC have been conducted on dairy cows [11], beef heifers [39], dairy goats [40], sheep [29] and red deer [41].

Respiration chambers have several advantages. They can be used to describe diurnal CH_4_ emission patterns by recording real-time CH_4_ flux during a few minutes and with repeated measurements for 24 h a day for a few days to estimate the mean daily CH_4_ production. This provides insight into underlying mechanisms of enteric CH_4_ formation and could reflect the immediate feedback from feed nutrition values or feed additives. Furthermore, it facilitates investigating the relationships between CH_4_ production and the effects of animal, feed intake, dietary chemical composition and feeding regime [10,29,34,42]. Major strengths of RCs also include the ability to make accurate measurements of emissions including CH_4_ from ruminal and hindgut fermentation—which would not be measured by many other methods—such as SF_6_ tracer technique, GF, sniffer method, LMD, head box or facemask, etc. Typically, there are also feed intake facilities, as well as feces and urine output and milk collection facilities in RCs. It thus provides an opportunity to examine gross energy intake and energy outputs in milk, feces, urine and CH_4_ in investigating energy partitioning, which will provide critical information in estimating feed energy values and in evaluating animal energy utilization efficiency [35,43,44]. Moreover, combining rumen fermentation parameters and rumen microbial analysis with CH_4_ emission measurement can illustrate insights of CH_4_ production mechanisms and improve the understanding of relationships between methanogenesis and rumen microbes [40,45].

There are also some limits to the usefulness of the RC technique. The weaknesses are that RC involves technically demanding analytical equipment and is expensive to construct and maintain. In addition, there are constraints on the number of animals that can be experimented upon at any single time. Furthermore, animals require training and acclimatization to withstand enclosure in RCs before being assessed, which requires high labor input and time cost. A greater concern is that measurements of CH_4_ production are made under artificial conditions on animals that are restricted in their movements [31,38]. CH_4_ emissions and feed intake values gathered under such artificial conditions may not reliably reflect those from the same animal actively grazing a pasture under highly variable environmental conditions (e.g., temperature fluctuations, wind chill, sporadic or heavy levels of rainfall and an uneven distribution of vegetation), [46] and the maximum intakes for animals are generally lower in RC than in grazing because of the restriction of animal movements [36,47]. There is also concern that milking, cleaning, sampling and feeding of animals while confined in an RC can interrupt the measurement of gases when staff members enter. The disturbing time thus needs to be discarded from the 24 h dataset to exclude the interruption of gas composition in RC [48,49]. Furthermore, Gardiner et al. [50] validated six RC facilities at five leading agricultural research centers around the UK and the uncertainty associated with the RC reduced from 25.7% to 2.1% after validation. The measurement uncertainty was mainly derived from the sample ducting and flow measurement. Therefore, system calibration and recovery tests should be carried out just before and after each experiment to ensure a recovery rate of CH_4_ as close to 100% as possible [29,41].

### 2.2. Sulfur Hexafluoride Tracer Technique

Sulfur hexafluoride tracer technique is a non-isotopic tracer method that has been used to measure CH_4_ production from indoor and grazing ruminants [13,14,15]. Sulfur hexafluoride is an inert gas, which is not produced naturally by ruminants and does not affect the normal processes of the rumen. The technique involves orally dosing a controlled release small brass permeation tube filled with liquid SF_6_ into the animal’s rumen [47]. A Teflon disk within the brass screw cap of the permeation tube ensures that the SF_6_ is released at a relatively stable rate, which is calibrated prior to insertion and after collection of each tube in the rumen [13]. A halter modified with a capillary tube is placed over the nose of the animal and connected to an evacuated collar, worn around the animal’s neck. As the vacuum within the canister decreases, SF_6_ and CH_4_ emissions from around the mouth and nostrils of the animal are sampled into the capillary tube, which is then transferred and stored in the collar. The release of SF_6_ provides a means of accounting for the dilution of gases near the mouth [13]. After removal from the animal, the canister contents are analyzed using gas chromatography for CH_4_ and SF_6_ concentration determination [31]. The ratio between the CH_4_ and SF_6_ concentrations in the exhaled and eructated gas mixture within the canister is used to calculate the CH_4_ emission rate within the collection period according to QCH_4_ = QSF_6_ × CH_4_C/SF_6_C, where QCH_4_ is the CH_4_ emission rate (mol/d); QSF_6_ is the SF_6_ release rate (mol/d) from the permeation tube; and CH_4_C and SF_6_C are the CH_4_ and SF_6_ concentrations (parts per trillion, ppt) as measured by gas chromatography. This original equation from Johnson et al. [13] took no account of any background CH_4_ and SF_6_ concentrations in the immediate environment, which are important factors in the accuracy of this technique [15,51,52]. To address this issue, separate equipment is deployed within the confines of the experimental area but, crucially, not in the immediate vicinity of the experimental animals. This additional equipment measures the ambient concentrations of CH_4_ and SF_6_ on each day of a study. The modified equation is CH_4_ (g/d) = (CH_4_C − CH_4_B)/(SF_6_C − SF_6_B) × SF_6_Q × MW CH_4_/MW SF_6_, where CH_4_C = CH_4_ concentrations measured in the canister (µg/m^3^); SF_6_C = SF_6_ concentrations measured in the canister (µg/m^3^); CH_4_B = CH_4_ concentrations measured in the ambient canister (µg/m^3^); SF_6_B = SF_6_ concentrations measured in the ambient canister (µg/m^3^); SF_6_Q = SF_6_ release rate from the inserted permeation tubes (g/d); MW CH_4_ = CH_4_ molecular weight and MW SF_6_ = SF_6_ molecular weight [15].

The SF_6_ technique has a number of advantages in that it does not require animal confinement, it is relatively inexpensive to implement, and it is relatively non-invasive. This makes it uniquely appropriate and valuable for studying CH_4_ emissions from a large number of individual grazing animals simultaneously. In the midst of the competing arguments about the relative accuracy and precision of the SF_6_ technique, it remains an accepted method to estimate CH_4_ emissions usually in nominal 24 h periods [13,53].

The SF_6_ technique does have disadvantages. A key assumption of the SF_6_ technique is that both of the SF_6_ and CH_4_ fluxes are identical through the nose and mouth of the animal. However, this assumption has been challenged as it has been shown, through RC studies, that CH_4_ production by cattle can vary throughout the day, usually peaking post-feeding, whereas SF_6_ diffuses at a constant rate [54]. Experiments comparing RC and the SF_6_ technique showed that CH_4_ emissions could be overestimated using the SF_6_ technique if the deployment of permeation tubes was prolonged [55]. Therfore, it is crucial to do recovery tests for the permeation tubes and to estimate the SF6 release rate just after the end of each study [56]. In addition, the correction of ambient gas concentrations in well-ventilated conditions, such as grazing, is usually minor. However, during stable nighttime conditions, wind velocities are low and this may result in inadequate mixing of the gases and hence inaccuracies in the data [57]. Lassey et al. [15] pointed out that the position of ambient air sampling within animal sheds could significantly affect the results of CH_4_ emission rates, since the concentrations of CH_4_ and SF_6_ in the shed could be accumulated unevenly. Although the average CH_4_ emissions from sheep measured by SF_6_ technique agree with RC, the relationship between each other is weak [14]. It is therefore necessary to narrow and balance the SF_6_ permeation rates in different experimental treatments for a strengthened relationship between CH_4_ estimation and SF_6_ release [54]. Particular attention should also be paid to the size, weight and wearability of the SF_6_ equipment in order to appreciate animal welfare and behavior [58]. In addition, the SF_6_ measurements should be adjusted by adding CH_4_ emissions from the rectum, which is 3% of the total CH_4_ emissions from the mouth, nostrils and rectum, in order to obtain the whole digestive tract CH_4_ production [59].

### 2.3. GreenFeed

GreenFeed (C-Lock Inc, Rapid City, SD, USA) is an automated head-chamber system combined with a portable feeding station for spot sampling of CH_4_ emissions and gaseous exchange in ruminants [60,61]. This system integrates the measurements of gas concentrations, airflow, bait feed intake and automated recognition of animal identification through a radiofrequency identification ear tag as animals approach the bait feed [16]. A gas sampling system is automated based on when an animal eats the feed in GF. The system sucks air through the animal’s nose and mouth into a duct with airflow measured. Then a subsample is drawn into a gas analysis system, where CH_4_ concentration is determined using a non-dispersive infrared sensor [62]. Gas concentrations of an animal are usually measured a few times a day within 3–7 min each time by controlling the feed supply in GF for a few days. A set of GF system is designed to measure as many as 20 animals [58]. The data of each individual animal collected in a few days are then used to calculate average daily CH_4_ emissions [17]. The program installed in GF controls the timing and amount of feed availability for each animal and distributes the measurements evenly in a 24 h feeding cycles [63,64]. Data are uploaded to a cloud-based analysis system in real-time developed by the GF manufacturer for CH_4_ emission estimation [17,38].

One advantage of GreenFeed is that it provides an alternative as a portable and automated technique in estimating individual animal’s CH_4_ flux under both indoor and grazing conditions. Reliable results can be obtained if the timing and times of each animal measurement are well controlled, which is easily achievable by the operation of an investigator in a tie stall barn [65]. GreenFeed is capable of differentiating the higher emitters from the lower ones in dairy cows and beef heifers as RC [39,66]. Huhtanen et al. [67] reported that CH_4_ production measured by GF was significantly correlated with that by RC (R^2^ = 0.92) in direct comparisons and also in line with CH_4_ emissions calculated by prediction equations developed by RC data.

A disadvantage of GreenFeed is that it has high between-day and between-animal variations. Hammond et al. [17] found that the GF technique didn’t detect the effects of diet and animal factors on CH_4_ emissions when compared with RC and SF_6_. This is possibly due to the requirement of a bait feed supplement to encourage the animal to use the facility, which may not be consumed equally by different animals and will interact with the dietary treatments, thus introducing between-day and between-animal variation in the measurement [9,58]. When used for animals freely grazing on pasture, it is voluntary for the animals to be assessed which could limit the measurement timing and frequency of individual animals and unbalance the number of animals measured in different treatment groups [17]. In addition, wind direction and speed changes could also impact measurement, which are major variation factors for grazing studies using GF [62,68]. Furthermore, CH_4_ emissions are strongly corelated with feed intake and form a clear diurnal rhythm in a 24 h feeding cycle [17,34]. Therefore, many studies highlighted the importance of controlling number and timing of GF visits per animal to ensure sufficient numbers of measurements throughout the 24 h feeding cycle to obtain accurate estimates of daily CH_4_ emissions [64,69,70]. Arbre et al. [71] suggested that a repeatability of 70% in CH_4_ yield (g/kg dry matter intake) measurement required 17 day periods and the repeatability could further increase to 90% up until 45 day periods when using the GF system. Therefore, the successful application of this technique relies on a sufficient number of animals, measurement periods and animal visits to GF.

### 2.4. Sniffer Technique

This method was developed by Garnsworthy et al. [18] on the basis of sampling and measuring gas concentration in the eructations of lactating dairy cows during milking. It is based on a hypothesis that there is close relationship between daily CH_4_ production and CH_4_ concentration in eructations and the associated eructation frequency. In this method, gases are continuously sampled into a polyethylene sampling tube installed in the feed trough of an automatic milking system when the cows are eating and being milked. The other end of the sampling tube is connected to an infrared CH_4_ concentration analyzer. Following this principle, Garnsworthy et al. [18] also developed a calculation index of daily CH_4_ emissions based on the mean peak area of CH_4_ concentration and the peak frequency in the eructations of dairy cows, which was further illustrated by Bell et al. [72].

One advantage of the Sniffer technique is that it could measure CH_4_ concentrations from a large number of individual lactating dairy cows repeatedly and rapidly during routine milking under commercial conditions. CH_4_ emission rate measured by the sniffer method during milking was linearly correlated with the CH_4_ production measured in RC (R^2^ = 0.79) [18]. Estimation of daily CH_4_ emissions using the sniffer method also agreed well with the daily CH_4_ emissions predicted using milk yield and body weight of dairy cows [19]. This technique could also detect high and low CH_4_ emitting cows as RC [72].

A disadvantage of the sniffer technique is that it exhibited a greater difference in between-cow and within-cow variability than the RC and SF_6_ techniques [18,72]. It is also reported to be less accurate in estimating CH_4_ production than the GF system. The accuracy of the sniffer technique is influenced by the uncertainties of dairy cow head movements in the feed trough, the various designs of feed trough and the sampling point positions. All of these factors may result in different air-mixing conditions and different dilution effects of ambient air on the gas concentration in eructations [62,73]. Wu et al. [73] found that these systematic errors disturbed the correlation of CH_4_ production rate with CH_4_ concentration measured by the sniffer method and could not be compensated by repeated measurements. In addition, the sniffer method does not actually measure CH_4_ flux or CH_4_ production. It only provides prediction values of CH_4_ emissions by CH_4_ concentrations from existing regression equations developed using RC [18,19,72]. Therefore, different equations may be required for different dietary scenarios.

### 2.5. Ventilated Hood

A ventilated hood or respiration head box is designed as a simplified RC using similar principles in gas measurement which covers only the animal’s head instead of its whole body [20,21]. The ventilated hood provides enough space for the movement of the animal’s head and free access to feed and water. In order to minimize the air leakage, a sleeve is attached with the hood at the animal’s neck position. There is also an air circulation system inside the hood and a pipe connected at the top of it which continuously sucks gases out to a multi-gas analyzer for concentration measurement. This also helps to maintain a slightly negative pressure in the ventilated hood to ensure minimum air leakage through the animal’s neck position.

The hood system provides a lower cost option with comparable measurement accuracy to RC. Furthermore, it is also able to reflect the real-time flux changes and diurnal variation of CH_4_ emissions, offering an opportunity to evaluate the immediate effects of possible CH_4_ mitigation strategies [21]. There was good agreement between the CH_4_ production values of individual finishing beef cattle measured by a hood system and their subsequent daily CH_4_ outputs by RC [74]. The mean CH_4_ yield in dairy cows obtained in a head box type system was also in line with those reported in RC but with a 70% lower cost in construction [20].

As with RC, animals are also restrained and require training to adapt to the ventilated hood or head box systems [36,58]. It only measures CH_4_ emissions from mouth and nostrils, as do the SF_6_, GF and sniffer techniques without accounting for the hindgut CH_4_ emissions [59].

### 2.6. Facemask

A facemask is another technique using a similar mechanism of gas concentration analysis to that of a ventilated hood and RC but in a manner of spot sampling [22,23]. The mask fully covers the muzzle by a strap attached around the neck of the animal. Gas sampling was performed by a tube that connected the mask to a mass flow controller and then gas analyzers [23]. The animal is usually confined within a squeeze chute to assist the measurement and the measurement typically lasts for 30 min and is done every 2–3 h, for a maximum of seven times a day [22]. The measurement frequency could further reduce to only once a day at 6 h after morning feeding for 2–3 days [22,23] since there is evidence that the sampling conducted at that time is strongly correlated with total daily CH_4_ emissions [75].

A facemask is much cheaper and portable when compared to an RC or a ventilated hood. It is also simpler than other methods (e.g., SF_6_ and GF) which facilitates screening more animals. Additionally, it can provide flux data if flow meters are included in the equipment design. The short-term (30 min/day for 3 days) facemask technique generated CH_4_ measurements that were comparable to those estimated using SF_6_ and RC across a range of dry matter intake levels in bulls [22]. It was also confirmed that the CH_4_ emissions measured using the facemask technique were comparable to those measured by RC at the same time point (6 h after morning feeding, 30 min/day for 2 days) in dairy cattle [23].

Compared with RC and ventilated hood methods, it requires more cooperation from the animal and restricts the animal from eating and drinking while the facemask is on during the measurement period. Furthermore, animals may feel uncomfortable when confined in a squeeze chute, which can interrupt the measurement procedure. Because CH_4_ emissions fluctuate over time, the timing and quantity of measurements relative to the diurnal patterns of CH_4_ emissions may have considerable impact on the results. Oss et al. [22] pointed out that the facemask method may have limitations in terms of assessing enteric CH_4_ mitigation strategies that are applied over a short duration to low numbers of animals due to higher animal-to-animal and day-to-day coefficients of variation. Silveira et al. [23] reported that a linear bias was detected in the relationship of the CH_4_ outputs between facemask and RC, indicating sources of variation such as feeding regime and CH_4_ emission fluctuation may limit the accuracy of the facemask method. Therefore, meaningful CH_4_ emission measurements are usually difficult and might lead to erroneous results [31,36].

### 2.7. Laser CH_4_ detector

Laser CH_4_ detector (Tokyo Gas Engineering Solutions Inc., Tokyo, Japan) is a hand-held device that can remotely measure CH_4_ concentrations in the air between the LMD and the muzzle of the animal using the infrared absorption spectroscopy technique [24,76]. The distance between the LMD and the animal is in a range of 1 to 3 m and the measurement period is typically between 2 to 4 min each time [38,58]. The unit of the CH_4_ concentration is then displayed as parts per million-meter (ppm-m). The LMD can normally be operated in an environment of −17 °C to 50 °C with 30% to 90% relative humidity [77]. The LMD equipment is originally applied in the detection of CH_4_ accumulation in industry areas such as coal mines, landfills and CH_4_ leakage in natural gas transmission pipelines, etc.

The LMD technique offers a convenient and inexpensive opportunity in measuring CH_4_ concentrations in ruminants and also possibly in evaluating CH_4_ mitigation strategies. The process of the LMD measurement is nonintrusive to the test animals with no adverse effect on their normal welfare and behavior. Chagunda et al. [24] observed a significant and positive relationship between the CH_4_ measurements with LMD and RC and confirmed that the estimates of CH_4_ emissions using LMD were sufficiently close with those of RC [78]. The LMD is also able to discriminate between differences in mean CH_4_ concentrations produced by different cow activities [25].

However, a major issue with this technique (similar to the sniffer technique) is that it only measures concentration and not flux. Obviously, there is a negative correlation between the concentration and the airflow rate as CH_4_ emissions are the product of these two values. Ricci et al. [76] reported that the correlation between LMD and RC was not consistent in different experimental periods when evaluating the LMD in estimating CH_4_ emissions from ewes and steers. In particular, the most accurate estimations from LMD were located 3 to 5 h post feeding [76]. Thus, it is necessary to integrate the effects of feeding regime and animal behavior on eructation and respiration in the assessment of LMD measurement results. Rey et al. [79] reported that the measurements of LMD were not as repeatable as those of the sniffer technique which was probably due to the much longer distance between the device and the animals in LMD. This consequently introduced more variation sources such as wind direction and speed and adjacent animals’ behavior and respiration. Moreover, other infrared absorbing compounds (e.g., water vapor in the air) can also affect the results. Similarly, particular attention should also be paid to changeable weather conditions when using LMD outdoors and in pastures for grazing animals, as variation in relative humidity, atmospheric pressure and temperature may limit the potential application of LMD [25]. Pickering et al. [80] tried to use LMD measurement results in screening the genetic trait of CH_4_ production in dairy cows. However, the repeatability within lactation was only 0.07 and across lactations was only 0.03. Therefore, it is still not fully qualified in the genetic evaluation of animals regarding CH_4_ emissions, unless further research is carried out to improve its repeatability.

### 2.8. Portable Accumulation Chamber

Portable accumulation chamber (PAC) is developed as a RC system without air circulation, which has been used as a simplified, fast, unexpansive and short-term method in estimating CH_4_ emissions for breeding values in sheep [81]. Goopy et al. [26] first designed a PAC system to estimate the CH_4_ production rate in sheep taken directly from the paddock, which is a transparent polycarbonate booth with a volume of around 0.8 m^3^. A sheep is sealed in the PAC for a maximum 2 h period while CH_4_ accumulates. The CH_4_ emissions during this enclosure period are calculated by measuring the CH_4_ concentration and then multiplying the net chamber volume [82]. CH_4_ concentration (optional together with other gases, e.g., CO_2_, O_2_ and NH_3_ if analyzers are available) could be measured every 30 min, after entering the PAC using gas analyzers with the ambient CH_4_ concentration corrected [81,83]. The net chamber volume is calculated as the volume of PAC minus that occupied by the animal which is estimated as 1 L/kg body weight [83].

Goopy et al. [26] demonstrated a 71% correlation coefficient between the CH_4_ emissions estimated by 1 h PAC measurements and those quantified by RC. Furthermore, the daily RC CH_4_ production could be repeated by three 1 h PAC measurements [84]. Therefore, short-term PAC measurements could be potentially applied in the evaluation of CH_4_ production levels in a large number of individual animals.

There is still uncertainty in the qualification of PCA in daily CH_4_ production estimation. It also only measures the concentration and not the flux of CH_4_, similar to the sniffer and LMD techniques. Hegarty [82] thought it was still not possible to scale the PCA CH_4_ emission rate up to daily CH_4_ production, especially without knowing the feed intake before short-term measurements. Robinson et al. [27] found that the low CH_4_ producing sheep screened by PAC did not produce low CH_4_ when tested by RC. The CH_4_ emission values estimated by PAC and RC didn’t agree well with each other, with the correlation coefficients as low as 0%–19%. Jonker et al. [81] further investigated that genetic correlations for CH_4_ emissions in sheep between measurements by RC and PAC were also low, indicting different operating conditions or different aspects of genetic traits that were examined by these two measurement methods. Therefore, concerns still exist over the capability of PAC measured data in estimating daily CH_4_ emissions with possible variations from inadequate data collection during short periods.

## 3. General Discussion

Above all, it is not possible to use one method for all conditions to reliably measure CH_4_ emissions. Each of the methods discussed in the current review have their unique scope of applications, advantages and disadvantages. A summary of comparison of enteric CH_4_ emission measurement techniques is shown in Table 1. In general, RC, SF_6_ tracer and ventilated hood are capable of continuous 24 h measurements of CH_4_ flux for each individual animal, providing accurate reference methods used for research and inventory purposes. In fact, the Intergovernmental Panel on Climate Change (IPCC) recommends using RC and head enclosures (e.g., ventilated hood or head box) for measuring the CH_4_ conversion factor (Y_m_), which is defined as the percentage of gross energy intake converted to CH_4_ [85]. Both RCs and head enclosures facilitate the feed intake measurement simultaneously, which is vital in calculating Y_m_ and is recommended as the Tier 2 methodology by IPCC for inventory reporting. Ultimately, CH_4_ is derived from the rumen fermentation of feedstuff and intake level explains most of the variation in CH_4_ production [86]. Therefore, CH_4_ measurements alone are of relatively little value without knowledge of feed intake. RC could provide real-time results of the enteric CH_4_ produced in the whole digestive tract, which is widely recognized as the gold standard in daily CH_4_ quantification. However, RC and head enclosures require relatively high labor input, time cost and animal training with a relatively low number of animal throughput. Furthermore, they are suitable for indoor use only. In contrast, SF_6_ tracer, GF and LMD techniques have advantages that apply to outdoor or grazing systems. However, reliable and accurate measurements of feed intake for outdoor or grazing animals is quite challenging. Although markers such as chromium sesquioxide (Cr_2_O_3_) [87], ytterbium oxide (Yb_2_O_3_) [88] and n-alkanes [89] have been used to estimate feed intake in studies which aimed to measure CH_4_ emissions by grazing cattle, the accuracy and precision are not comparable with direct measurement in RC. All short-term methods (i.e., GF, sniffer, facemask, LMD and PAC) introduce additional sources of variation including numbers and timing of measurements obtained relative to the 24 h feeding cycle. Therefore, short-term measurements can be meaningful only if a sufficient number of animals are examined with the measurements distributed across various representative times of the day over a long enough period, and if a good relationship with RC measurements can be obtained. In particular, GF could measure CH_4_ flux, which provides important airflow data that are not available in sniffer, LMD and PAC methods. Meanwhile, it has much less interruption on animal behavior and welfare compared to the facemask method. There still needs to be considerable improvement in the reliability and repeatability of sniffer, LMD and PAC when using their short-term concentration measurements to predict CH_4_ production. However, the low-cost and simplicity of their application makes the short-term measurements suitable for a large number of measurements in individual animals under their practical production conditions. This offers a potential opportunity in defining the CH_4_ phenotype required for genetic and genomic improvement for breeding lower emitting animals. Last but not least, the CH_4_ emissions from hindgut fermentation (3% of that from the whole digestive tract [59]) should be added in the results of SF_6_, GF, sniffer, ventilated hood, facemask and LMD measurements because the animal’s whole body is not sealed in these systems as it is when using the RC and PAC methods.

## 4. Conclusions

In conclusion, correct and successful use of CH_4_ emission measurement methods relies on the optimum matching between the objectives of the studies and the mechanism of each method. Respiration chambers and head enclosures are accurate enough for determining emission factors for IPPC inventory reporting, however they are not possible for use in grazing animals. Sulphur hexafluoride tracer technique is able to be applied in grazing situations, however the herbage feed intake relies on indirect prediction. The short-term techniques (i.e., GF, sniffer, facemask, LMD and PAC) provide potential opportunities in identifying high and low CH_4_ emitters in a large group of animals for breeding purposes, although future research is still needed to improve their reliability and repeatability. Overall, ideal CH_4_ measurement techniques should be accurate, rapid, cost effective and automated with an appreciation of animal behavior and welfare that enables measurement of animals under their practical production environment.

## Figures and Tables

**Table 1:** Comparison of enteric CH_4_ emission measurement techniques.

Method	Indoor/Grazing	CH_4_/Multi-Gas	Rumen/Hindgut	Continuous/Short-Term	Flux/Concentration
Respiration chambers	Indoor	CH_4_, multi-gas	Rumen, hindgut	Continuous	Flux
Sulphur hexafluoride tracer	Indoor, grazing	CH_4_	Rumen	Continuous	Flux
GreenFeed	Indoor, grazing	CH_4_, multi-gas	Rumen	Short-term	Flux
Sniffer method	Indoor	CH_4,_ multi-gas	Rumen	Short-term	Concentration
Ventilated hood	Indoor	CH_4_, multi-gas	Rumen	Continuous	Flux
Facemask	Indoor	CH_4_, multi-gas	Rumen	Short-term	Flux
Laser CH_4_ detector	Indoor, grazing	CH_4_	Rumen	Short-term	Concentration
Portable accumulation chamber	Indoor	CH_4_, multi-gas	Rumen, hindgut	Short-term	Concentration
